# Pharmacologically-induced mitotic synchrony in airway epithelial cells as a mechanism of action of anti-inflammatory drugs

**DOI:** 10.1186/s12931-015-0293-4

**Published:** 2015-10-29

**Authors:** R J Freishtat, G Nino, Y Tsegaye, S E Alcala, A S Benton, A M Watson, E K M Reeves, S K Haider, J M Damsker

**Affiliations:** Division of Emergency Medicine, Children’s National Health System, Washington, DC USA; Department of Pediatrics, George Washington University School of Medicine and Health Sciences, Washington, DC USA; Department of Integrative Systems Biology, George Washington University School of Medicine and Health Sciences, Washington, DC USA; Division of Pulmonary and Sleep Medicine, Children’s National Health System, Washington, DC USA; George Washington University School of Medicine and Health Sciences, Washington, DC USA; Children’s National Health System, Washington, DC USA; ReveraGen Biopharma, Inc., Silver Spring, MD USA

**Keywords:** Anti-inflammatory, Asthma, Epithelium, Glucocorticoid

## Abstract

**Background:**

Mitotic synchrony is the synchronous progression of a population of cells through the cell cycle and is characteristic of non-diseased airway epithelial cells. However, we previously showed that asthmatic airway epithelial cells are characterized by mitotic asynchrony and are pro-inflammatory as a result. Glucocorticoids can induce mitotic synchrony that in turn suppresses the pro-inflammatory state of diseased cells, suggesting a novel anti-inflammatory mechanism of action. Herein, we benchmarked traditional glucocorticoids against the ability of a new clinical-stage dissociative steroidal drug, VBP15, for mitotic resynchronization and associated anti-inflammatory activity in asthmatic airway epithelial cells.

**Methods:**

Primary airway epithelial cells differentiated at air-liquid interface were exposed to VBP15, dexamethasone or vehicle following in vitro mechanical injury. Basolateral cytokine secretions (TGF-β1, IL-6, IL-10, IL-13, and IL-1β) were analyzed at different time points using cytometric bead assays and mitosis was examined by flow cytometry.

**Results:**

VBP15 improved mitotic synchrony of proliferating asthmatic cells in air-liquid interface cultures compared to vehicle-exposed cultures. VBP15 also significantly reduced the basolateral secretion of pro-inflammatory (i.e. IL-1β) and pro-fibrogenic cytokines (i.e. TGF-β1) in air-liquid interface-differentiated asthmatic epithelial cultures following mechanical injury.

**Conclusion:**

VBP15 improves mitotic asynchrony and injury-induced pro-inflammatory and fibrogenic responses in asthmatic airway epithelial cultures with efficacy comparable to traditional glucocorticoids. As it is predicted to show superior side effect profiles compared to traditional glucocorticoids, VBP15 holds potential for treatment of asthma and other respiratory conditions.

## Background

Glucocorticoids are well-established as a front-line therapy in asthma. However, there are persistent concerns about their long-term side-effects (e.g. growth failure in children; adrenal suppression; weight gain) [1]. VBP15 is a clinical-stage dissociative glucocorticoid identified as a lead compound in a screening program focused on Δ-9,11 glucocorticoid-analogues that have potential as safer alternatives to traditional glucocorticoids [2–5]. These Δ-9,11 compounds are differentiated from glucocorticoids by the key conversion of a hydroxyl group to a carbon-carbon double bond that retains transrepression activities (NFκB inhibition), while reducing transactivation activities (GRE-mediated gene transcription) [6]. VBP15 showed efficacy in mouse models of Duchenne muscular dystrophy and multiple sclerosis while showing a much reduced side effect profile when benchmarked against prednisolone [2, 5].

In the context of asthma, we recently showed that oral VBP15 abrogates pulmonary eosinophilia in murine ovalbumin-induced allergic pneumonitis with comparable efficacy to the traditional glucocorticoid, prednisone [3]. These data suggest that VBP15 is an anti-inflammatory alternative to traditional oral glucocorticoids for acute or chronic asthma. However, immune-mediated inflammation is only one component of asthmatic inflammation. Structural airway cells, such as epithelial cells, are also important contributors to the pathogenesis of asthma [7]. In fact, we and others have shown that asthmatic epithelium is intrinsically inflammatory via secretion of cytokines [8, 9]. We further demonstrated that asthmatic airway epithelial inflammation is secondary to the abnormal mitotic behaviors of its regenerative basal cell population [10]. Specifically, nonasthmatic airway epithelial basal cell populations progress in relative synchrony through the cell cycle (G_1_, S, G_2_/M), while asthmatic epithelial basal cells proliferate with a more even distribution among the cell cycle phases (i.e. mitotic asynchrony) [8, 10]. In other words, at any given time, the majority of the epithelial progenitors in non-asthmatic airways can be found in a single cell cycle phase. Whereas asthmatic epithelial progenitors are distributed more evenly through all of the cell cycle phases. This mitotic asynchrony induces basolateral secretion of inflammatory and fibrogenic cytokines. Using mitotic capture (i.e. pause mitosis at a single checkpoint temporarily) with pulse-exposure to a traditional glucocorticoid (i.e. dexamethasone), we resynchronized asthmatic epithelial mitosis, thereby reducing the associated cytokine secretion [8, 10]. Herein we test the hypothesis that, similar to traditional glucocorticoids, VBP15 also reduces asthmatic airway epithelial-derived inflammation.

## Methods

### Cell culture and intermittent drug exposures

Non-asthmatic and asthmatic airway epithelial cells (#AIR-606 and #AIR-606-Asthma; MatTek Corp., Ashland, MA) were grown and differentiated at air-liquid interface using previously described protocols [8, 11, 12]. Briefly, these epithelial cells were proliferated on 6-well collagen-coated Transwell membrane plates. The airway epithelial cells were maintained at 37 °C for 7 days and then placed at air-liquid interface with proprietary basolateral media (provided by the manufacturer; MatTek Corp.) lacking glucocorticoids and epidermal growth factor. Basolateral media was replaced every 48 h for 21 days until the epithelial cells differentiated as confirmed visually by microscopy. After differentiation, the epithelial cultures were exposed to VBP15 (*n =* 3), dexamethasone (DEX) (*n =* 6) or dimethyl sulfoxide (DMSO) vehicle for 2 h at −26, −2, +22, and +46 h. Cultures were mechanically scrape-wounded at 0 h and thereafter exposed continuously to bromodeoxyuridine (BrdU) to identify mitotically-active cells, as further detailed below. All experiments were determined to be exempt from the requirement of Institutional Review Board review and approval under United States Department of Health and Human Services exemption 2 of section 46.101(b) of 45 Code of Federal Regulations 46.

### Mechanical injury model

**Fig. 1:**
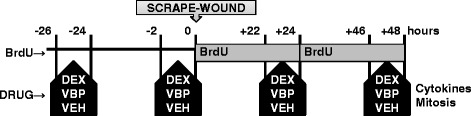
Experimental design for in vitro wounding of airway epithelial cultures: Epithelia were pulsed for two hours every 24 h with 20nM dexamethasone (DEX), 10 μM VBP15 (VBP), or vehicle (VEH) at the times shown. Mechanical scrape-wounding occurred at zero hours with continuous BrdU exposure until cell harvest at +48 h for cytokine and mitotic analyses. Media samples were frozen before measurement of inflammatory cytokines

A previously described in vitro airway epithelial injury model for studying epithelial repair processes in the lung was used in this study [8, 13–15] (Fig. 1). Briefly, using a p1000 pipette tip, epithelial cultures were scraped in two perpendicular lines at 0 h. The epithelial cultures were then placed in BrdU-containing (10 μM) medium which was replaced after a 2 h pulse of either VBP15 (10 μM), DEX (20 nM), or DMSO vehicle at +24 h. Epithelial cultures were incubated at 37 °C and 5 % CO_2_ until +48 h after the injury. Cells were exposed to trypsin for 5 min at 37 °C and resuspended in trypsin neutralization solution to obtain a single cell suspension.

**Fig. 2:**
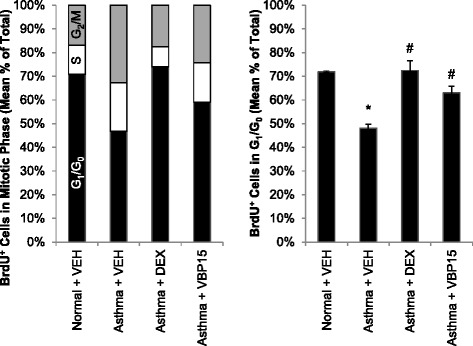
VBP15 Improves Mitotic Asynchrony in Asthmatic Human Airway Epithelia: Compared with wounded non-asthmatic epithelial cultures, wounded asthmatic epithelial cultures at +48 h showed a more even distribution of BrdU^+^ cells among the cell cycle phases (i.e., G_1_/G_0_, S, and G_2_/M) consistent with mitotic asynchrony (Left Panel). Pulse exposures of asthmatic epithelial cultures to VBP15 improved cell cycle synchrony as shown by normalization of the percentage of mitotic cells in each cell cycle phase although to a lesser extent than for dexamethasone (DEX)-exposed epithelial cultures. Statistical comparisons of the percentage of BrdU^+^ cells in G_1_/G_0_ among conditions are shown (Right Panel). Experiments were conducted in triplicate in *N =* 3 donors per group. (*p < 0.05 vs. normal vehicle, ^#^ *p <* 0.05 vs. asthma vehicle)

**Fig. 3:**
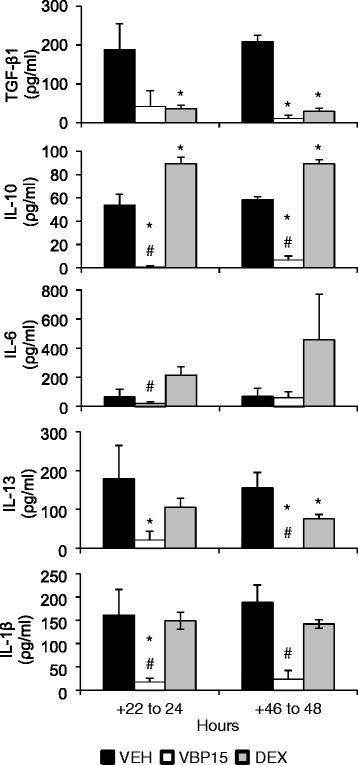
VBP15 effectively reduces post-wounding asthmatic epithelial basolateral inflammatory secretions: Levels of cytokines measured by cytometric bead assay are shown for basolateral secretions from asthmatic and non-asthmatic human airway epithelial cells differentiated at air-liquid interface. Measurements were obtained post-injury at +24, and +48 h. Data are shown as mean ± SEM in pg/mL and significance was obtained by *t*-test using corresponding time points for DMSO vehicle (VEH)-exposed, DEX-exposed, and VBP15-exposed asthmatic cultures. Experiments were conducted in triplicate using *N =* 3 donors. (*p < 0.05 vs. vehicle, ^#^ *p <* 0.05 vs. DEX)

### Analysis of inflammatory mediators and cell cycle analysis

Cytometric bead arrays (BD Biosciences, San Jose, CA) were used to measure inflammatory (IL-1β, IL-6, IL-10, and IL-13) and fibrogenic (TGF-β1) cytokines in apical and basolateral secretions at 0, +24, and +48 h. These cytokines were selected as all, but IL-6, were significantly altered between asthmatic and non-asthmatic cultures in our prior study [8]. Although we did not observe significant differences in IL-6 in that study, IL-6 levels trended higher in the asthmatic cultures at many time points. Further, IL-6 is receiving increasing attention as a potential mediator of the marker of airway epithelial cell activation [16]. For these reasons, we continue to include IL-6 in our standard cytokine assays.

For cell cycle analyses, the cells were simultaneously labeled at +48 h with the following according to the manufacturers’ protocols: (1) Carboxyfluorescein FLICA Apoptosis Poly-Caspase Detection Kit (Immunochemistry Technologies, LLC, Bloomington, MN) and (2) APC BrdU Flow Kit containing 7-amino-actinomycin-D (BD Biosciences, San Jose, CA). Data were analyzed using the cell cycle analysis feature of FlowJo 7.6 (Tree Star, Inc., Ashland, OR).

### Statistical analyses

Statistical comparisons were performed in SAS 9.3 software (SAS Institute Inc., Cary, NC) using *t*-test functions of log-transformed data within time points. Results are reported as mean ± SEM unless otherwise noted. Significance level was p ≤ 0.05.

## Results

### VBP15 improves mitotic synchrony in asthmatic human airway epithelium

We first examined the effects of VBP15 in restoring mitotic synchrony during the regenerative phase in asthmatic airway epithelial cell cultures. In these experiments, human airway epithelial cells underwent mechanical injury (wounding) as previously described [8], and then were harvested into single-cell suspensions at +48 h for flow cytometry to characterize cellular distribution in G_1_/G_0_, S, and G_2_/M, focusing on cells incorporating BrdU. Of note, there was no significant difference in level of BrdU incorporation between the VBP15 and DEX culture conditions for wounded asthmatic epithelial cultures (mean ± SEM: 0.10 ± 0.04 % versus 0.10 ± 0.04 % of total cells; *P =* 0.91).

Mitosis in non-asthmatic wounded airway epithelial cultures was synchronous (e.g. >70 % of cells in G_1_/G_0_) and unaltered by the administration of DEX. In contrast, wounded asthmatic epithelial cultures exhibited mitotic asynchrony relative to non-asthmatic control cultures (G_1_/G_0_, S, G_2_/M: 47 ± 0, 21 ± 2, 33 ± 2 % for asthmatic airway epithelial cells vs. 71 ± 1, c-asthmatic airway epithelial cells; Fig. 2). Mitotic synchrony was improved in wounded asthmatic airway epithelial cultures exposed to VBP15 (59 ± 4, 17 ± 1, 24 ± 3 %), but not to the same degree as DEX (74 ± 3, 8 ± 3, 18 ± 0 %).

### VBP15 reduces post-wounding asthmatic epithelial basolateral inflammatory and fibrogenic secretions

Given the observed beneficial effect of VBP15 on epithelial mitotic synchrony, we next tested whether VBP15 also abrogates post-wounding asthmatic epithelial basolateral inflammatory and fibrogenic cytokine secretions (i.e. IL-1β, IL-6, IL-10, IL-13 and TGF-β1). Previous studies have shown that these cytokines play an important role in asthmatic inflammation and airway remodeling [17]. Consistent with our previous experiments, human asthmatic epithelial cultures secreted significantly more basolateral cytokines following wounding relative to non-asthmatic preparations. DEX pulses altered the secretion of various inflammatory and fibrogenic cytokines (reduced TGF-β1 and IL-13; increased IL-10) in a similar manner to our previously published results [8].

There were similarities and differences between DEX and VBP15 in terms of effects on basolateral cytokine secretion. Similar to DEX, VBP15 reduced TGF-β1 secretion by approximately 80 % at +24 h and 95 % at +48 h (*p <* 0.05; Fig. 3^3^). As well, IL-13 secretion was reduced approximately 50 % at +48 h by DEX and by VBP15, almost to zero. In addition, VBP15 significantly reduced the secretion of IL-10, IL-6, and IL-1β while DEX increased their secretion (i.e. IL-10) or had no effect (i.e. IL-6, IL-1β). Baseline values of cytokines were consistent with vehicle control values which remained unchanged through the course of the experiment.

## Discussion

There is a critical need to develop new asthma medications that retain the highly beneficial action of traditional glucocorticoids, but without the deleterious effects of their long-term use [1]. Herein, we studied VBP15, a novel Δ-9,11 glucocorticoid-analogue that optimizes several activities of traditional glucocorticoids while minimizing side effects [2–5], in an in vitro model of human airway epithelia lacking immune cells. Our results are the first to demonstrate that VBP15 improves mitotic asynchrony and inflammatory/fibrogenic cytokine secretion in asthmatic human airway epithelial cells.

VBP15 is a dissociative glucocorticoid identified as a lead compound in a screening program focused on Δ-9,11 glucocorticoid-analogues [2–5]. VBP15 possesses a molecular modification in the 9,11 position of the traditional glucocorticoid ring structure which results in dissociation of glucocorticoid-mediated anti-inflammatory signaling (transrepression, NFκB inhibition), from glucocorticoid-receptor-mediated transcriptional activity and subsequent side-effects (transactivation) [2–4]. We have recently confirmed that VBP15 exhibits robust in vivo anti-inflammatory activity mediated by inhibition of NFκB activation that is at least as potent as that seen with traditional glucocorticoids [2, 3]. We have also shown that this mechanism occurs independently of glucocorticoid response element (GRE) activation (transactivation), or upregulation of inhibitory transcripts [2]. As GRE-regulated genes have been implicated in traditional glucocorticoid deleterious effects, including osteopenia, stunted growth and adrenal suppression, Δ-9,11 glucocorticoid-analogues are particularly promising because they provide a clear approach to improve traditional glucocorticoid side effect profiles.

We have recently reported that VBP15-mediated inhibition of NFκB is sufficient to retain the anti-inflammatory activity of conventional glucocorticoids [2]. Importantly, despite the lack of GRE-mediated transcriptional properties, systemic VBP15 treatment is at least as effective as prednisolone, a traditional glucocorticoid treatment, at reducing pulmonary NFκB activity, leukocyte degranulation, and the release of Th2 pro-inflammatory cytokines (i.e. IL-13) in the lungs of a murine model of ovalbumin-induced allergic asthma [3]. Moreover, VBP15 treatment does not result in stunting of growth [2, 3]—a major known side-effect of glucocorticoids in childhood asthma [1].

Initial experiments evaluated whether VBP15 restores mitotic synchrony in ALI-differentiated asthmatic airway epithelial cell cultures. This is a relevant question for the asthmatic condition because epithelial mitotic asynchrony is an important biological feature of the disease [8, 10, 18]. Importantly, similar to the effects seen with the traditional glucocorticoid, dexamethasone, VBP15 improved epithelial mitosis in asthmatic airway epithelial cells and promoted appropriate airway epithelial mitotic synchrony after mechanical injury. The latter findings may be connected to our recent data demonstrating that VBP15 has beneficial physicochemical effects on the plasma membrane, protecting cells from injury and promoting membrane repair [2].

Extended experiments in asthmatic airway epithelial cells exposed to VBP15 identified that the intrinsic secretion of inflammatory and fibrogenic cytokines was abrogated by VBP15 to an even greater degree than dexamethasone. In concert with the apparently better mitotic synchrony-inducing effect of dexamethasone, this raises the possibility of a threshold effect rather than a linear dose–response between mitotic synchrony and cytokine secretion. In addition, IL-10 was actually decreased by VBP15 in these experiments even though dexamethasone increases the expression of this important anti-inflammatory cytokine. This supports the need for future investigation as to whether the beneficial reductions in pro-inflammatory cytokines outweigh potentially harmful reductions in anti-inflammatory cytokines.

This study is an initial step to examine the possible therapeutic actions of VBP15 on the human respiratory epithelium, a potential target for the treatment of asthma, and we acknowledge several limitations. Although we used three different human airway epithelial donors, there is still the possibility of significant heterogeneity among the individuals with regard to asthma phenotype, treatment regimens and severity of disease at the time of tissue availability. In addition, our data do not directly acknowledge the long term remodeling consequences implicated by our model as they show the relation between inflammation and mitotic asynchrony in response to acute injury. Thus, different models are needed to specifically investigate the effect of chronic airway damage and the in vivo effect of VBP15 in other cells implicated in airway remodeling, including sub-epithelial fibroblasts and airway smooth muscle. In addition, we cannot make any conclusions about the potencies nor the physiological relevance of the concentrations used for VBP15 versus dexamethasone. Lastly, in evaluating the implications of the present study, it must be emphasized that our observations pertain to studies conducted in an in vitro human airway model lacking inflammatory cells. Therefore, the extent to which these observations relate to the human condition is open to debate.

## Conclusions

In summary, VBP15 improves mitotic asynchrony and injury-induced pro-inflammatory and fibrogenic responses in asthmatic airway epithelial cultures with efficacy comparable to traditional glucocorticoids. Together with our prior in vivo data and the predicted superior side effect profiles, VBP15 holds potential for treatment of asthma and other respiratory conditions.
